# Peroxiredoxin 5 Acts as a Negative Regulator of the Sodium-Chloride Cotransporter Involved in Alleviating Angiotensin II-Induced Hypertension

**DOI:** 10.3390/antiox14010100

**Published:** 2025-01-16

**Authors:** Hoon-In Choi, In Ae Jung, Soo Wan Kim

**Affiliations:** Department of Internal Medicine, Chonnam National University Medical School & Hospital, Gwangju 61469, Republic of Korea; hoonin_c@hanmail.net (H.-I.C.); inae673@naver.com (I.A.J.)

**Keywords:** peroxiredoxin 5 (Prdx5), Angiotensin II (Ang II), sodium-chloride cotransporter (NCC), hypertension, chronic kidney diseases (CKD)

## Abstract

Chronic kidney disease (CKD) and hypertension are interconnected, worsening each other. Recent studies have shown that the reduction of peroxiredoxin 5 (Prdx5) accelerates kidney fibrosis, a hallmark of CKD. This study aims to observe whether the deficiency of Prdx5 also contributes to the worsening of CKD-related hypertension. Angiotensin II (Ang II, 1000 ng/kg/day) was infused into Prdx5 wild-type (WT) and Prdx5 knock out (KO) mice (each group; n = 6). The blood pressure was higher in the Ang-II-infused Prdx5 KO mice than in the WT mice. Ang-II-induced ROS/RNS generation and fibrotic marker expressions were also higher in the Prdx5 KO mice. In particular, the expression of the sodium-chloride cotransporter (NCC), an ion transport protein important for sodium retention in the distal convoluted tubule, and the NCC’s phosphorylation at Thr53 were increased in the kidney of Ang-II-infused Prdx5 KO. The activity of an WNK4-SPAK/OSR1, upstream activator of the NCC, was also increased. In 209/mDCT cells, the knockdown of Prdx5 (siPrdx5) increased the activity of Ang-II-mediated WNK4-SPAK/OSR1-NCC signaling and Ang-II-mediated ROS generation, whereas Prdx5 overexpression showed opposite results. In conclusion, Prdx5 negatively regulates the WNK4-SPAK/OSR1-NCC signaling axis, indicating its potential as a candidate for antihypertensive drug development through NCC regulation.

## 1. Introduction

Hypertension and kidney disease have similar pathophysiology and are bidirectionally related [1,2,3]. In a large chronic kidney disease (CKD) cohort study, increased extracellular fluid (ECF) volume was identified as an independent determinant of uncontrolled hypertension and resistant hypertension in CKD patients [4]. Hypertension in CKD patients indicates impaired renal pressure–natriuresis mechanisms, resulting from sodium retention accompanied by ECF volume expansion [5,6]. There are several factors that modulate renal urinary sodium excretion, including the sympathetic nervous system (SNS), renin–angiotensin system (RAS), aldosterone, and insulin. Angiotensin II (Ang II) is a major effector of the RAS, is involved in the pathogenesis of renal fibrosis leading to end-stage renal disease, and plays a role as a major determinant in the pressure–natriuresis responses [7,8]. However, further studies on the cellular and molecular mechanisms linking the correlation between Ang-II-mediated renal fibrosis and Ang-II-mediated hypertension are still needed.

The distal convoluted tubule (DCT) reabsorbs approximately 5–10% of the filtered sodium load [9]. The sodium-chloride cotransporter (NCC) is expressed only in the DCT and is the target for effective antihypertensive therapeutics, such as thiazide diuretics. Since the NCC is important in fine tuning salt homeostasis, alterations in NCC function significantly impact blood pressure regulation. The importance of NCC expression and activity in blood pressure regulation has been elucidated via the discovery of monogenic mutations associated with hypertension. Gitelman syndrome, a loss of NCC function, is characterized by hyponatremia and low blood pressure [10,11]. Gordon’s syndrome, also known as pseudohypoaldosteronism type II, is characterized by gain-of-function mutations in genes encoding NCC regulators and is a disorder that results in salt retention and hypertension [12,13,14]. In the DCT, the function of NCC is mainly regulated by two processes. One of the processes is the regulation of NCC in the apical membrane of the DCT. The other process is regulating the activity of NCC by the phosphorylation of threonine residues 55 and 60 and serine 73 in humans (threonine 53 and 58 and serine 71 in mice and rats). These phosphorylation sites are key residues in the amino-terminal domain [15,16]. Two related serine–threonine kinases directly phosphorylate NCC, STE20/SPS1-related proline/alanine-rich kinase (SPAK) and oxidative-stress-responsive kinase 1 (OSR1). In the kidney, lysine deficient protein kinases (WNKs) are known activators of SPAK/OSR1 and mediate activation through phosphorylation SPAK at Thr 243 or Ser 383 and OSR1 at Thr 185 or Ser 325. Among WNKs, it has been reported that Ang-II-mediated NCC activation is a WNK4-dependent process via phosphorylating SPAK and OSR1 [17,18]. Although there are limitations in the development of therapeutics for hypertension, the possibility of a therapeutic strategy targeting the regulatory pathway, WNK4-SPAK/OSR1, as a master activator of NCC is promising [19].

Peroxiredoxin 5 (Prdx5) is an atypical member of the peroxiredoxin family that reduces not only hydrogen peroxide but also peroxynitrite [20]. It is localized in the cytoplasm, nucleus, mitochondria, and peroxisomes and performs specific functions depending on its intracellular localization. In a recent study, a reduction or ablation of Prdx5 in the kidney was reported to be a pathophysiological phenomenon that increased the sensitivity to ischemia/reperfusion (I/R)-induced acute kidney injury and unilateral-ureteral-obstruction-induced renal fibrosis [21,22,23]. In particular, in kidney-specific tubule cells, either rat kidney interstitial fibroblast cells (NRK49F) or mouse distal convoluted cells (209/mDCT), Prdx5 had an anti-fibrotic effect on transforming growth factor-β (TGF-β)-induced fibrosis in a Cys48-dependent manner [22,23].

In this study, a hypertensive mouse model was prepared via long-term infusion of Angiotensin II (Ang II) in Prdx5-knock-out (KO) mice. For the first time, we investigated whether renal Prdx5-deletion accelerated the progression of renal fibrosis, a common feature in CKD, and whether Prdx5-deletion promoted the progression of hypertension. Next, we determined whether the deletion of Prdx5 was related to the expression and activity of NCC, which has been shown to cause hypertension, and whether the efficacy of Prdx5 was dependent on the WNK-SPAK/OSR1 pathway, the master activator of NCCs.

## 2. Materials and Methods

### 2.1. Animals

The CRISPR/Cas9-mediated Prdx5 KO mice (C57BL/6N-Prdx5em1Gmcr, Endonuclase-mediated mutation 1, Deletion) were produced by GEMCRO Inc. (Seoul, Republic of Korea) with Toolgen’s technology on a C57BL/6N background. The gene editing removed exons 3–6 of the *Prdx5* gene (accession number NM_012021), which left the *Trmt112* gene sequence region unaffected. The prepared F1 mice were crossed and maintained. The animal experiments were approved by the Animal Care Regulations (ACR) Committee of Chonnam National University Medical School (CNUHIACUC-21062). Our protocols conformed to the institution’s guidelines for experimental animal care and use. The experiments were performed using male Prdx5 KO mice, and wild-type littermate mice were used as the control (8 ~ 12 weeks old). The mice were housed under controlled temperature (21 ± 2 °C) in a 12-h light–dark cycle. The genotyping primer information and PCR conditions to distinguish between WT and Prdx5 KO mice are as follows: common forward primer: 5′-CACCCTTAGGTCCAAGCTTCC-3′; WT reverse primer: 5′-TCGACCCCACTCTTCAATCAC-3′; KO reverse primer: 5′-CCAAATCCCTGCCACTGACT-3′. PCR conditions: 95 °C (5 min)/ 95 °C (30 s), 65 °C (30 s), 72 °C (30 s); 33 cycles; 72 °C (5 min); 4 °C (ꝏ). WT and KO were confirmed to have a size of 264 bp and 327 bp, respectively.

### 2.2. Reagents and Antibodies

Angiotensin II (Ang II, Cat# A9525) and dihydroethidium (DHE, Cat# D7008) were purchased from Sigma (St. Louis, MO, USA). The osmotic minipump was purchased from ALZET (Cat#2004, Cupertino, CA, USA). The 4-hydroxy-2-hexenal (HHE) antibody was purchased from COSMO BIO CO., LTD. (Tokyo, Japan). Antibodies against SPAK, WNK4, vimentin, and the HA tag were purchased from Cell Signaling Technology (Danvers, MA, USA). OPN was purchased from Santa Cruz (Dallas, TX, USA). Fibronectin was purchased from BD Biosciences (Franklin Lakes, NJ, USA). NCC and p-SPAK (Ser373)/p-OSR1 (Ser325) were purchased from Millipore (Burlington, MA, USA). Phospho-NCC (Thr53) was purchased from Phosphosolution (Aurora, CO, USA). Antibodies against α-SMA, GAPDH, and β-actin were purchased from Sigma-Aldrich (St. Louis, MO, USA). DMPO and CPH were purchased from Enzo Biochem (Farmingdale, NY, USA). The specific antibody against Prdx1~6, Trx1, and TR was a gift from Dr. Ho Zoon Chae (Chonnam National University, Gwangju, Republic of Korea).

### 2.3. Ang-II-Infused Hypertension Model

Male Prdx5 WT and Prdx5 KO mice at 8~10 weeks of age (n = 6, each group) were used to develop the hypertensive model via subcutaneous infusions of Ang II (Sigma-Aldrich, St. Louis, MO, USA) at a dose of 1000 ng/kg/min or saline using osmotic mini-pumps (Alzet MODEL 2004; Lot # 10389-18; DURECT, Cupertino, CA, USA) with a flow rate of 0.23 μL/h for four weeks [24]. After inserting the osmotic mini-pump, the blood pressure was measured by using volume pressure recording (VPR), a tail-cuff method of CODA noninvasive blood pressure system (Kent Scientific, Torrington, CT, USA), according to the manufacturer’s instructions every week for four weeks [25]. In brief, mice were induced to freely enter the holder, restricted movement, and acclimatized to a pre-warmed thermo-regulator for at least 5 min. The acclimation cycle was set to 5, and the cycles per set were set to 20 cycles before measurement. For the obtained data, only data marked as true in both regular cycle and accepted were used, and measurement values with a standard deviation greater than 30 were not used. After four weeks, blood and urine (spot urine) were collected, and serum and urine chemical parameters (sCr, BUN, sNGAL, urinary PCR, and urinary ACR) were analyzed. Tissue from the kidney was fixed in 10% formalin for IHC or frozen in optimal cutting temperature (OCT) compound (Leica Biosystems, Richmond, IL, USA) for the preparation of tissue slides. In addition, the tissue was rapidly cooled in liquid nitrogen and stored at −70 °C for protein expression, mRNA expression, and other experiments. Experiments to compare Prdx5 hetero mice were conducted following the same procedure as the Prdx5 KO experiment set, except that each group had n = 5.

### 2.4. Cell Culture and Ang II Treatment

Mouse distal convoluted tubule cells (209/mDCT; ATCC, Manassas, VA, USA) were cultured in a 1:1 ratio mixture of Dulbecco’s Modified Eagle Medium (DMEM) supplemented with 1 g/L glucose and 1 mM sodium pyruvate (Life Technologies, Cat#11885: Carlsbad, CA, USA) and Ham’s F-12 Nutrient Mix (Life Technologies Cat# 11765). The complete medium was supplemented with 5% fetal bovine serum (FBS), 50 U/mL penicillin, and 50 μg/mL streptomycin at 37 °C in a humidified 5% CO_2_ incubator. For the transient expression of Prdx5 in 209/mDCT cells, HA-tagged mouse wild-type (WT) Prdx5 was used. Plasmid DNA constructs were transfected into 209/mDCT cells using Fugene HD transfection reagent (Promega, Madison, WI, USA) at a 1:3 ratio of DNA to transfection reagent. Twenty-four hours after the transfection, the cells were starved with serum-free media for one day, followed by treatment with Ang II (1 μM) for the indicated times.

### 2.5. Immunohistochemistry and Immunofluorescence

The kidney tissue was fixed in 10% formalin and then immersed in phosphate buffer saline (PBS). The tissue was embedded in paraffin and cut into 4-μm sections. Standard IHC protocol was followed after deparaffinization and rehydration. The specimens were incubated with the following primary antibodies: rabbit polyclonal antibodies directed against Prdx5 (diluted 1:1000) [23], rabbit polyclonal antibodies directed against fibronectin (diluted 1:200; BD Biosciences, Franklin Lakes, NJ, USA), and mouse monoclonal antibodies directed against α-SMA (diluted 1/500; Sigma Chemical Co., St. Louis, MO, USA). The primary antibodies were incubated at 4 °C overnight. The tissue specimens were subjected to PAS staining to evaluate the extent of the kidney tissue damage. The staining was developed with diaminobenzidine (DAB) chromogenic substrate (DakoCytomation, Glostrup, Denmark) and counterstained with hematoxylin. Randomly selected fields of the stained images were examined for Prdx5 (×200 or ×400), α-SMA (×200), and fibronectin (×200) staining. PSR staining was performed according to the manufacturer’s protocol. Mouse distal convoluted tubule cells (209/mDCT) were seeded onto four-well cell culture slides (2 × 10^4^ cells/well) and were processed as mentioned previously. The cells were washed with PBS and were fixed in 4% paraformaldehyde for ten minutes. Subsequently, the cells were permeabilized with permeabilization buffer (0.5% Triton X-100 in PBS), and the slides were incubated with primary antibodies for p-NCC (Thr53) (Phosphosolutions, Cat# p1311-53, 1/1000 dilution) diluted with the equilibration buffer (1% BSA, 0.5% Triton X-100 in PBS) at 4 °C overnight. Following incubation with the primary antibody, the cells were washed with the equilibration buffer and incubated for one hour at room temperature with anti-Rabbit Cy3-conjugated secondary antibodies (Abcam, Cambridge, UK). The nuclei were counterstained using SlowFade Gold antifade reagent with DAPI (Invitrogen, Waltham, MA, USA). The images were captured using a confocal microscope (LSM 510; Carl Zeiss, Oberkochen, Germany). The images were magnified at ×800, Bar = 50 μm.

### 2.6. Reactive Oxygen Species (ROS) Detection

For detection of lipid peroxidation products in the kidney tissue, specimens were incubated with mouse anti-HHE antibody (diluted 1/50; COSMO BIO Co., Ltd., Tokyo, Japan), and then tissue sections were incubated with the HRP-conjugated secondary antibody (diluted 1/50; Cell Signaling Technology, Danvers, MA, USA) for one hour at room temperature. Staining was developed with the DAB chromogenic substrate (DakoCytomation) and counterstained with hematoxylin. The frozen kidney tissue was cut into 8-µm slices and prepared on slides to measure the amount of superoxide anion in the kidney tissue. Specimen slides were rinsed with pure water for 30 s and immediately stained with 5 μM DHE for 20 min. The slides were then washed three times with pure water, mounted immediately, and DHE fluorescence (excitation 535 nm/emission 610 nm) was photographed with a fluorescence microscope (Nikon Eclipse Ni, Nikon: Tokyo, Japan) within 30 min. Randomly selected fields of the stained images were examined (×400; for HHE and DHE). To determine the species of direct free radicals of ROS and RNS induced by Ang II, DMPO and CPH (ENZO, Farmingdale, NY, USA) were used. Freshly prepared kidney proteins were reacted with 100 mM DMPO and 2 mM CPH at 37 °C for 20 min and then stored at −70 °C until analysis. The measurement of DMPO and CPH adducts was performed using a CW/Pulse EPR System (Korea Basic Science Institute, Seoul, Republic of Korea) with the following instrumental set-up: frequency of 9.86 GHz, power of 5 mW, modulation amplitude of 1 G, modulation frequency of 100 kHz, time constant of 20.48 ms, conversion time of 20.00 ms, sweep time of 24 s, and scan of 4. All measurements were performed at room temperature, and the data were collected by using the manufacturers’ software.

### 2.7. Quantification of mRNA

Total RNA was extracted from mice heart tissues using the TRIzol reagent (Invitrogen). The extracted RNA (1 μg) was reverse-transcribed into complementary DNA (cDNA) using a QuantiTect-Reverse Transcription Kit (Qiagen, Hilden, Germany). A quantitative real-time polymerase chain reaction (qRT-PCR) was performed using SyGreen Blue Mix Hi-ROX (PCRBIO-SYSTEMS, London, UK) and the StepOnePlus Real-Time PCR System (Thermo Fisher Scientific, Waltham, MA, USA). The primers used in qRT-PCR are listed in Table S1.

### 2.8. Western Blotting Analysis

To prepare the protein lysates, the tissue was homogenized in radioimmunoprecipitation assay (RIPA) buffer (50 mM Tris-HCl pH 7.6, 150 mM NaCl, 5 mM EDTA, 1% NP 40, 0.1% SDS, and protease inhibitors). Similarly, the cell lysates were also sonicated in RIPA buffer. The tissue and cell lysates were centrifuged at 15,000× *g* and 4 °C for 10 min to remove cell debris. Immunoblotting was performed, as described previously [23], to detect target proteins using the indicated antibodies. The protein band intensities were quantified using ImageJ software (version number 1.54f; National Institutes of Health, Bethesda, MD, USA).

### 2.9. Statistics

All statistical analyses were performed using GraphPad Prism 8 software, version 8.01 (GraphPad Software, Inc., San Diego, CA, USA). The Mann–Whitney test and Kruskal–Wallis test were used for single or multiple comparisons, and Dunn’s post hoc test was used to determine statistical significance. *p*-value less than 0.05 were considered to indicated significant differences.

## 3. Results

### 3.1. Prdx5 KO Mice Were More Susceptible to Ang-II-Induced Hypertension

**Figure 1:**
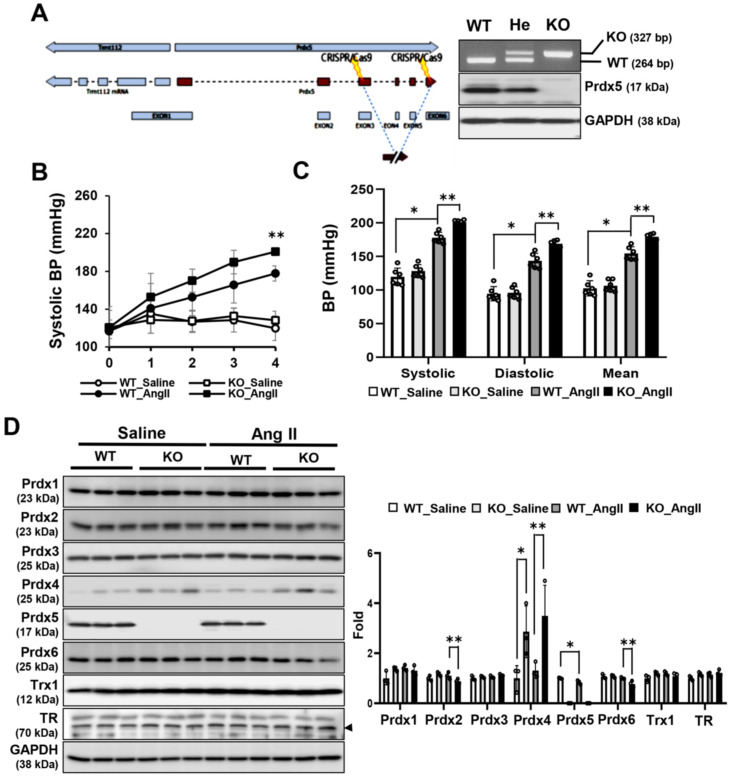
Elevated blood pressure in Ang-II-infused Prdx5 knock-out (KO) mice. (**A**) Prdx5 wild-type (WT), hetero (He), and knock-out (KO) mice were identified by genotyping as described in Materials and Methods. WT and KO were confirmed to have a size of 264 bp and 327 bp, respectively. In each mouse, the protein expression level of Prdx5 was confirmed in the kidney tissue. (**B**) Prdx5 WT and KO mice were infused with saline (WT_Saline and KO_Saline) or Ang II (WT_Ang II and KO_Ang II), and the blood pressure was recorded weekly for four weeks. Changes in systolic BP were observed for four weeks. (**C**) Systolic, diastolic, and mean BP at four weeks after Ang II infusion were compared between each group. (**D**) Expression change in Prdxs isotype, Trx1, and TR in Prdx5 KO mouse kidney were checked by Western blotting. GAPDH was used as an internal control. For bar grapes, the value of the WT_Saline group was set to one and expressed as a fold change. All values are presented as the mean ± SD. Statistical significance was measured using Mann–Whitney test. * *p* < 0.05, compared with the saline-infused Prdx5 WT. ** *p* < 0.05, compared with Ang-II-infused Prdx5 WT.

Prdx5 KO mice were used to observe the effect of Prdx5 deletion on Ang-II-induced hypertension (Figure 1A). Prdx5 KO mice were selected through genotyping, and it was confirmed that the mRNA and protein levels of Prdx5 were deleted in all tissues (Figure S1A,B). The systolic blood pressure of the Prdx5 WT and KO mice started to rise after one week of Ang II infusion and gradually increased until four weeks. At four weeks after Ang II infusion, the systolic, diastolic, and mean BP were all higher in the Ang-II-infused Prdx5 KO mice than in the Ang-II-infused WT mice (Figure 1B,C). Among the Prdxs isotypes, the expression of Prdx4 was increased in Prdx5 KO mice, and after Ang II infusion, Prdx4 was further increased, while Prdx2 and Prdx5 showed a pattern of decreased protein expression (Figure 1D). These results suggest that Prdx5 may be one of the targets involved in blood pressure maintenance.

### 3.2. Deletion of Prdx5 in the Kidney Exacerbates Ang-II-Induced Renal Injury

**Figure 2:**
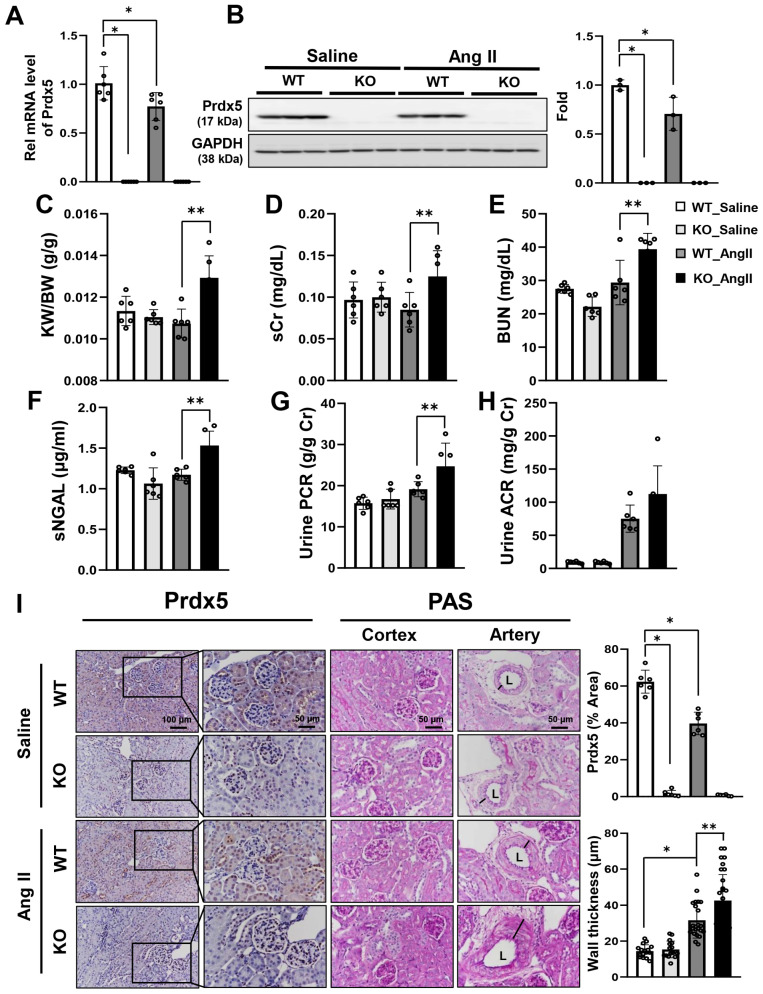
Effects of PRDX5 on Ang-II-mediated renal injury. The mRNA level (**A**) and protein level (**B**) of Prdx5 were checked. Renal functional parameters were checked to confirm whether Prdx5 was involved in Ang-II-induced renal injury. (**C**) Kidney weight to body weight (KW/BW, g/g) ratio. (**D**) Serum creatinine (sCr, mg/dL). (**E**) Blood urea nitrogen (BUN, mg/dL). (**F**) Serum neutrophil gelatinase-associated lipocalin (sNGAL, μg/mL). (**G**) Urine PCR (protein-to-creatinine ratio g/g Cr). (**H**) Urine ACR (albumin-to-creatinine ratio, mg/g Cr, *p* = 0.0588). (**I**) IHC staining of Prdx5 and PAS staining. Six randomly selected regions were used for quantification of IHC images. The wall thickness of the artery on the PAS was quantified by measuring the length in a blinded fashion for each group (N = 16–32). The L symbol indicates lumen. Scale bar indicates 100 μm (original magnification ×200) or 50 μm (original magnification ×400). All values are presented as the mean ± SD. Statistical significance was measured using Mann–Whitney test. * *p* < 0.05, compared with the saline-infused Prdx5 WT. ** *p* < 0.05, compared with Ang-II-infused Prdx5 WT.

The kidney is not only an important organ for blood pressure control but also a major organ affected by hypertension. Therefore, we determined whether the loss of Prdx5 influenced Ang-II-induced renal damage. It was confirmed that the expression of Prdx5 by Ang II was decreased at the mRNA and protein levels (Figure 2A,B). The kidney weight to body weight (KW/BW) ratio was increased in the Ang-II-infused Prdx5 KO mice, and the levels of kidney injury markers, such as serum creatinine (sCr), blood urea nitrogen (BUN), serum neutrophil gelatinase-associated lipocalin (sNGAL), urine PCR (protein to creatinine ratio), and urine ACR (albumin to creatinine ratio) were increased in the Ang-II-infused Prdx5 KO mice compared with the Ang-II-infused WT mice (Figure 2C–H). Histologically, Prdx5 expression was also decreased during 4 weeks of Ang II infusion, and PAS staining results showed that increased mesangial expansion of the glomerulus and decreased brush border at the proximal tubule were more observed in the kidneys of Prdx5 KO mice injected with Ang II than in WT mice injected with Ang II, and the thickness of the renal artery was also observed to be increased (Figure 2I). These results suggest that deletion of Prdx5 may be a factor in aggravating the kidney’s physiological and morphological abnormality caused by Ang II.

### 3.3. Ang-II-Derived ROS/RNS Generation Is Increased in the Kidneys of Prdx5 KO Mice

**Figure 3:**
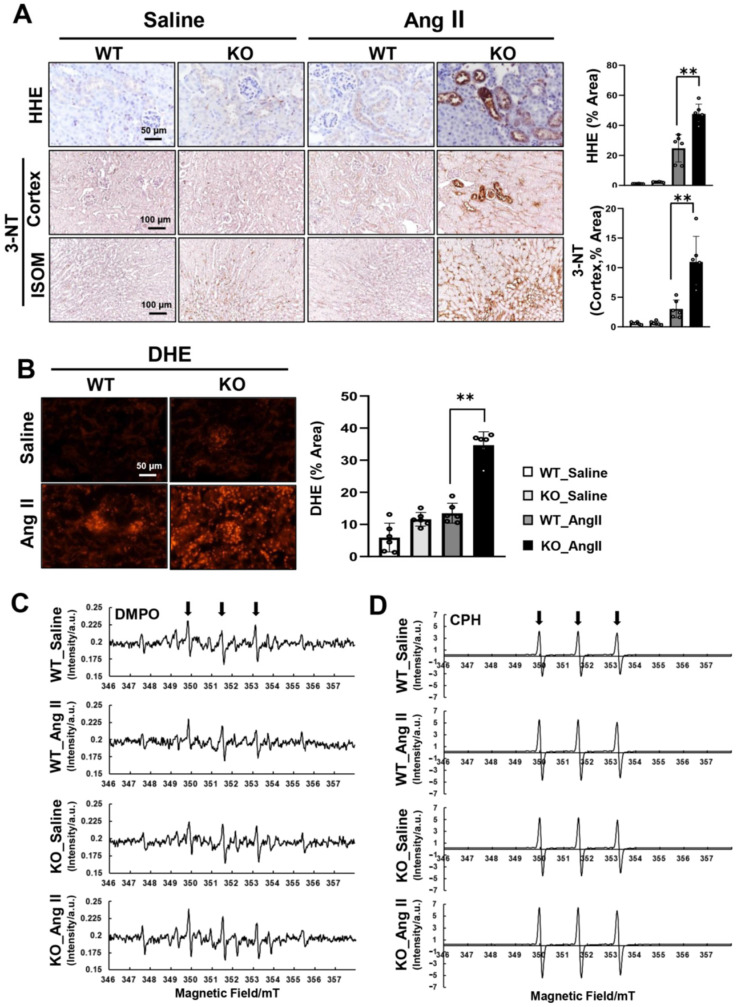
The increased ROS/RNS in Ang-II-infused Prdx5 KO kidney. (**A**) The increased HHE level and 3-NT level in Ang-II-infused Prdx5 KO kidney. Level of lipid peroxidation and protein oxidation was analyzed by immunohistochemistry with a specific anti-HHE antibody or anti-3-NT antibody, respectively. Image was magnified at ×400, Bar = 50 μm. (**B**) The increased superoxide anion level was analyzed in frozen mouse kidneys. DHE fluorescence (excitation 535 nm/emission 610 nm) was photographed with a fluorescence microscope (Nikon Eclipse Ni). Image was magnified at ×400, Bar = 50 μm. (**C**,**D**) Analysis of DMPO adduct and CPH adduct generated in Ang-II-infused Prdx5 KO kidney. Radicals trapped by DMPO and CPH were detected using electron paramagnetic resonance (EPR). All values are presented as the mean ± SD. Statistical significance was measured using Mann–Whitney test. ** *p* < 0.05, compared with Ang-II-infused Prdx5 WT.

There is accumulating evidence suggesting that Ang-II-induced oxidative stress plays a pivotal role in the pathogenesis of hypertension [26]. Prdx5 is an antioxidant enzyme that scavenges reactive oxygen species (ROS) and reactive nitrogen species (RNS). There was strong staining of 4-hydroxy-2 hexenal (HHE), a lipid peroxidation marker, in the renal tubule of Ang-II-infused Prdx5 KO mice. Furthermore, the expression of 3-NT, a protein oxidative biomarker, was also strongly expressed in the kidney cortex and ISOM of Ang-II-infused Prdx5 KO (Figure 3A). The level of superoxide anion in frozen kidney sections was analyzed by dihydroethidium (DHE) staining to confirm whether Prdx5 deficiency affected Ang-II-induced ROS generation. DHE fluorescence (excitation 535 nm/emission 610 nm) was increased in the Ang-II-infused kidney, and the staining was stronger in the Ang-II-infused Prdx5 KO kidney than in the Ang-II-infused WT kidney (Figure 3B). To directly identify the species of ROS and RNS generated by Ang II, radicals trapped by DMPO and CPH were observed using electron paramagnetic resonance (EPR). DMPO is a spin probe that traps superoxide anion and O-, C-, S-, and N-centered free radicals, and CPH is a spin probe that traps superoxide radicals and peroxynitrite. The peaks of DMPO adduct and CPH adduct were confirmed, and the intensity of each peak was confirmed to be increased in Ang-II-infused kidney of Prdx5 KO mice (Figure 3C,D). These results indicate that the species of ROS and RNS formed by Ang II are a type of superoxide and peroxynitrite, and that these radicals were further increased in the Prdx5 KO mouse kidney due to Prdx5 deficiency.

### 3.4. Prdx5 Negatively Regulates Ang-II-Induced Renal Fibrosis

**Figure 4:**
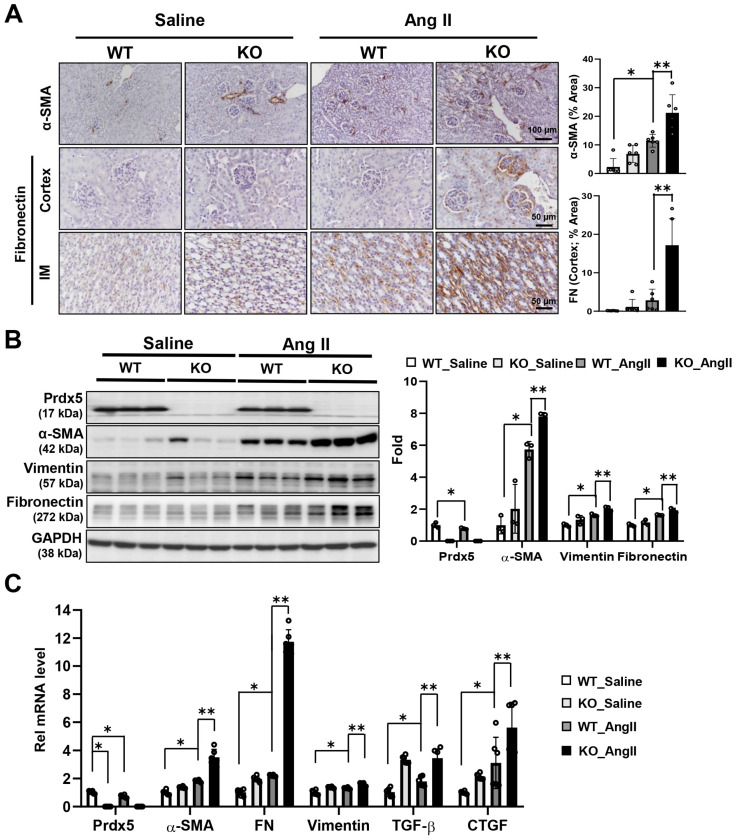
Accelerated progression of renal fibrosis in Ang-II-infused Prdx5 KO mice. (**A**) IHC staining of fibrotic markers. To confirm the effects of Prdx5 on the progression of Ang-II-induced renal fibrosis, α-SMA and fibronectin, known fibrotic markers, were identified on paraffin slides. The strongly stained cortex stained for α-SMA (×200, Bar = 100 μm) was imaged, and the cortex and inner medullar sections stained for fibronectin (×400, Bar = 50 μm) were imaged. Six randomly selected regions were used for quantification of IHC images. (**B**) Total protein expression of Prdx5 and the fibrotic markers. Changes in the total protein expression of Prdx5 and the fibrotic markers, α-SMA, vimentin, and fibronectin, were observed via Western blotting. GAPDH was used as an internal control. (**C**) Changes in the mRNA expression of Prdx5 and the fibrotic markers. For bar grapes, the value of the WT_Saline group was set to one and expressed as a fold change. All values are presented as the mean ± SD. Statistical significance was measured using Mann–Whitney test. * *p* < 0.05, compared with the saline-infused Prdx5 WT. ** *p* < 0.05, compared with Ang-II-infused Prdx5 WT.

To determine whether Prdx5 deficiency is associated with the aggravation of renal fibrosis by Ang II, the expression of fibrosis markers was examined. The expression of α-SMA was increased in the kidneys of Ang-II-infused Prdx5 KO mice compared with the Ang-II-infused WT mice. Fibronectin staining was increased in the glomerulus of Ang-II-infused Prdx5 KO mice, and fibronectin expression in the inner medulla was also increased (Figure 4A). Consistent with the immunohistochemical (IHC) results, the protein levels of fibrosis markers α-SMA, vimentin, and fibronectin were observed to be decreased in the kidneys of Ang-II-infused Prdx5 KO (Figure 4B). The mRNA level of the fibrotic markers was highest in kidneys from Ang-II-infused Prdx5 KO mice (Figure 4C). These results suggest that Prdx5 deficiency is associated with the exacerbation of Ang-II-induced renal fibrosis.

### 3.5. Deletion of Prdx5 Affects Ang-II-Induced Hyperactivity of the WNK4-SPAK/OSR1-NCC Signaling Axis

**Figure 5:**
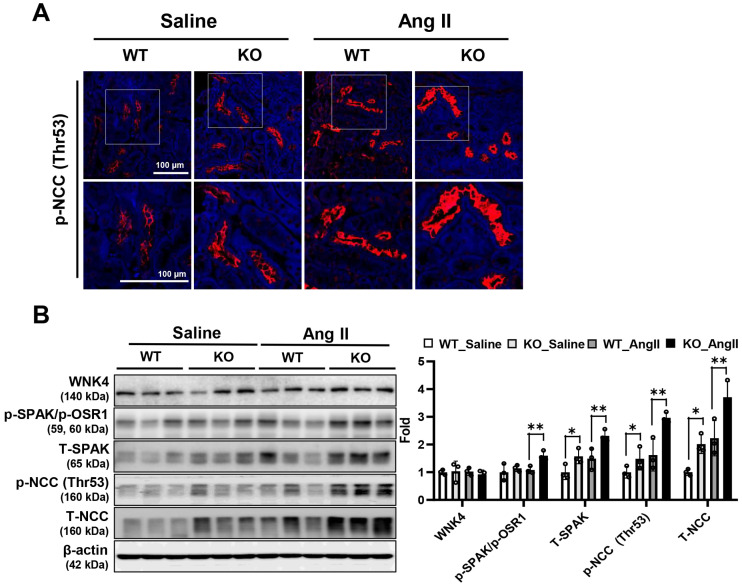
Increased NCC activation in Ang-II-infused Prdx5 KO kidney. (**A**) Activation of NCC. The kidney paraffin section was stained using an antibody that specifically detected NCC Thr53 phosphorylation. (**B**) Activation of the WNK4/SPAK/OSR-1/NCC signaling axis. The activity of the WNK4/SPAK/OSR-1 signaling pathway, which serves as the main axis of NCC activation, was examined. Changes in the expression level and the degree of WNK4/SPAK/OSR-1/NCC axis activation were confirmed using antibodies that detect the total and phosphorylated form. The value of the WT-saline group was set to one and expressed as a fold change. The β-actin was used as an internal control. All values are presented as the mean ± SD. Statistical significance was measured using Mann–Whitney test. * *p* < 0.05, compared with the saline-infused Prdx5 WT. ** *p* < 0.05, compared with Ang-II-infused Prdx5 WT.

Ang-II-infused Prdx5 KO mice showed increases in blood pressure, renal injury markers, ROS, and renal fibrosis. It could be hypothesized that Prdx5 is closely related to the regulatory mechanisms of Ang II. Next, we determined the mechanism of action in which the decrease in Prdx5 in the kidney affected the increased blood pressure. NCC is a thiazide-sensitive sodium-chloride cotransporter, which is an important pharmacological target for treating hypertension. NCC activity is activated by phosphorylation on key serine/threonine sites, and its activity is inhibited by downregulating the surface expression of NCC by ubiquitination and a series of endocytosis. First, to check whether there is a change in NCC activity, an immunofluorescent study was performed using an antibody specific for NCC Thr53 phosphorylation. As a result, it was observed that the amount of NCC Thr53 phosphorylation was increased in the apical membrane (Figure 5A). Next, the degree of activation of the WNK/SPAK/OSR1/NCC axis was confirmed in the whole kidney protein, and it was observed that the phosphorylation of NCC (Thr 53) was increased in Ang-II-infused Prdx5 KO. The phosphorylation of SPAK (Ser373) and OSR1 (Ser325), known as upstream regulators of NCC, also increased. However, the amount of WNK4, an upstream molecule of SPAK/OSR1, did not change between the Ang-II-infused Prdx5 WT and KO groups (Figure 5B). These results suggest that Prdx5 is involved in the activation of the WNK4-SPAK/OSR1-NCC axis by Ang II.

### 3.6. Prdx5 Negatively Regulates NCC Activity in a WNK4-SPAK/OSR1-NCC Signaling-Axis-Dependent Manner

**Figure 6:**
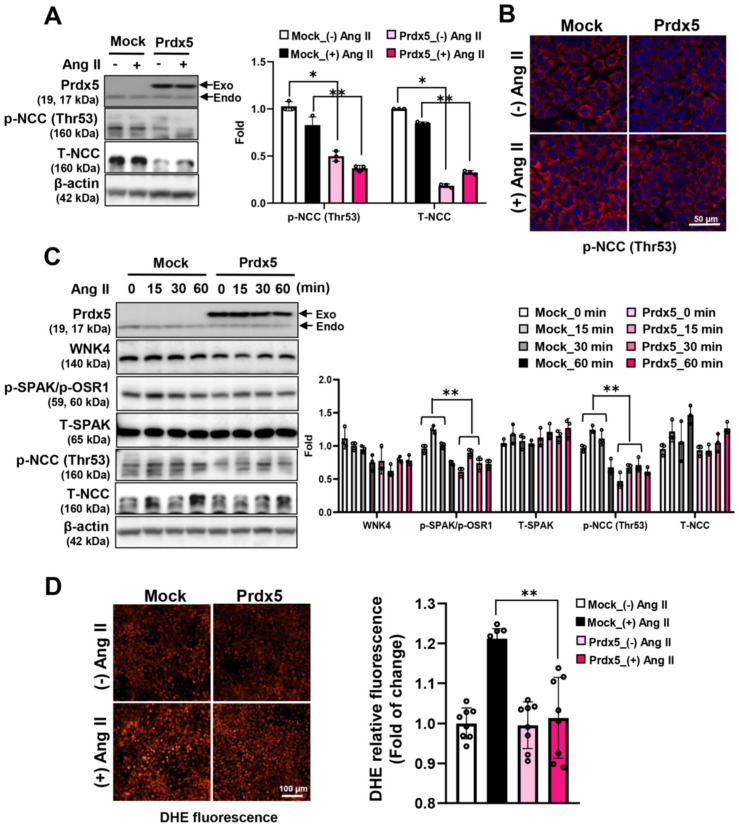
Downregulation of WNK4-SPAK/OSR1-NCC signaling by the overexpression of Prdx5. (**A**) Reduced expression and activity of NCC by the overexpression of Prdx5. HA-tagged Prdx5 was overexpressed in 209/mDCT cells to investigate the regulatory potential of Prdx5 on NCC. The cells were treated with Ang II (1 μM) for 24 h. The expression and activity of NCC were confirmed using an NCC antibody against the total and Thr53 phosphorylated form. (**B**) Immunofluorescence (IF) staining of the phosphorylated NCC (Thr53) via Prdx5. Overexpressed HA-tagged Prdx5 209/mDCT cells were seeded in four wells. After treatment with Ang II (1 μM) for 24 h, the phosphorylated NCC was visualized using a Cy3 fluorescence stain (Red). Images were visualized using confocal microscopy (magnification at ×800, Bar = 50 μm). (**C**) Prdx5 regulation of the WNK4/SPAK/OSR-1/NCC axis in Ang-II-treated 209/mDCT cells. Ang II (1 μM) was applied to 209/mDCT cells at the indicated times (0, 15, 30, and 60 min) to confirm the change in the activity of the signal molecules and to examine whether the activity of the WNK4/SPAK/OSR-1/NCC axis, an upstream signal pathway affecting the activity of NCC, was regulated via Prdx5. The β-actin was used as an internal control. The bar graph is displayed as a fold change with the value of untreated mock cells set to one. (**D**) The intracellular superoxide anion was labeled using the DHE probe. Images were immediately visualized using confocal microscopy (magnification at ×200, Bar = 100 μm) and analyzed using imageJ software. The value of the Mock was set to one and expressed as a fold change. All values are presented as the mean ± SD. Statistical significance was measured using Mann–Whitney test. * *p* < 0.05, compared with Ang II untreated Mock. ** *p* < 0.05, compared with Ang-II-treated Mock vs. Prdx5overexpression group in indicated time (0, 15, 30, and 60 min).

**Figure 7:**
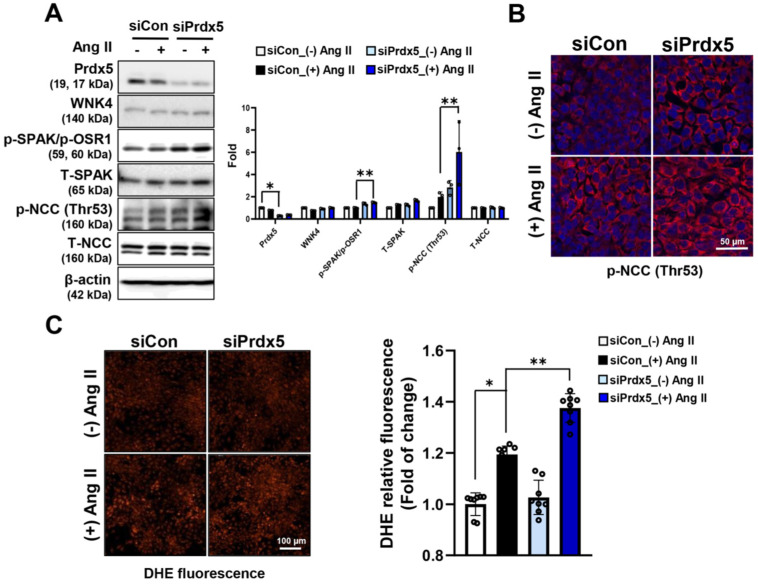
Hyperactivation of WNK4-SPAK/OSR1-NCC signaling by knockdown of Prdx5. To reconfirm the effect of Prdx5 downregulation on the WNK4-SPAK/OSR1-NCC axis activity, Prdx5 knockdown was induced in 209/mDCT cells using si-Prdx5. (**A**) After treatment with Ang II (1 μM) for 60 min, the level of total form and phosphorylated form on WNK4-SPAK/OSR1-NCC axis were confirmed by Western blotting. The β-actin was used as an internal control. The bar graph is displayed as a fold change with the value of untreated siCon cells set to one. (**B**) Immunofluorescence (IF) staining of the phosphorylated NCC (Thr53) by knockdown of Prdx5. After treatment with Ang II (1 μM) for 24 h, the phosphorylated NCC was visualized using a Cy3 fluorescence stain (Red). (**C**) The intracellular superoxide anion was labeled using DHE probe. Images were immediately visualized using confocal microscopy (magnification at ×200, Bar = 100 μm) and analyzed using imageJ software. The value of the siCon was set to one and expressed as a fold change. All values are presented as the mean ± SD. Statistical significance was measured using Mann–Whitney test. * *p* < 0.05, compared with Ang II untreated siCon. ** *p* < 0.05, compared with Ang-II-treated Mock vs. siPrdx5 knockdown group.

To further investigate the regulatory mechanism of Prdx5 in the WNK/SPAK/OSR1/NCC signaling axis, we transiently overexpressed HA-tagged Prdx5 WT protein in 209/mDCT cells, a mouse terminal curvature cell line, and examined the phosphorylation of NCC in response to Ang II treatment. As a result, the expression of the total and phosphorylated form (Thr53) of NCC were downregulated in 209/mDCT cells overexpressing Prdx5 (Figure 6A). Consistent with the changes in the protein expression level and activity of NCC, the activity of NCC by Ang II treatment was significantly reduced in Prdx5-overexpressing 209/mDCT cells compared with mock-transfected cells (Figure 6B). In Ang-II-treated 209/mDCT cells during the indicated time, the activity of WNK4/SPAK/OSR1, an upstream signaling pathway affecting NCC phosphorylation, was also downregulated by overexpression of Prdx5 (Figure 6C). In addition, it was also confirmed that the increase in ROS level by Ang II was effectively reduced in the cells overexpressing Prdx5 WT (Figure 6D). Conversely, in 209/mDCT cells with a Prdx5 knockdown by si-Prdx5, the phosphorylation of NCC (Thr53) was further increased after Ang II treatment. In addition, phosphorylation of SPAK/OSR1, an upstream molecule of NCC, was also significantly increased (Figure 7A,B). It was confirmed that the increase in ROS level by Ang II treatment was further aggravated by the decrease in the protein level of Prdx5 by si-Prdx5 (Figure 7C). Taken together, Prdx5 not only suppresses ROS production generated by Ang II treatment but also acts as a negative regulator of activation of the WNK4-SPAK/OSR1-NCC axis. Consequently, it can be considered that Prdx5 plays an important role in maintaining blood pressure via NCC regulation.

### 3.7. Prdx5 Hetero Mice Were Also More Susceptible to Ang-II-Induced Hypertension, the Mechanism of Which Also Involved Activation of NCC

**Figure 8:**
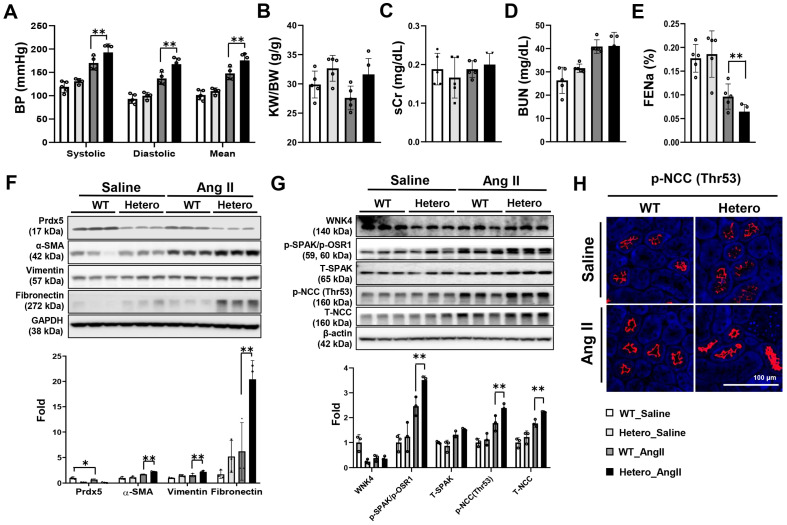
Increased blood pressure in Ang-II-infused Prdx5 hetero mice and its mechanism through NCC activation. (**A**) The change in blood pressure in the Prdx5 hetero mouse by Ang II infusion for 4 weeks. Renal functional parameters were checked in each group. (**B**) Kidney weight to body weight (KW/BW, g/g) ratio. (**C**) Serum creatinine (sCr, mg/dL). (**D**) Blood urea nitrogen (BUN, mg/dL). (**E**) Fractional excretion of sodium (FENa; %). (**F**) Changes in the total protein expression of PRDX5 and the fibrotic markers, α-SMA, vimentin, and fibronectin, were observed via Western blotting. GAPDH was used as an internal control. (**G**) Activation of the WNK4/SPAK/OSR-1/NCC signaling axis. Changes in the expression level and the degree of WNK4/SPAK/OSR-1/NCC axis activation were confirmed using antibodies that detect the total and phosphorylated form. The β-actin was used as an internal control. The value of the WT-saline group was set to one and expressed as a fold change. (**H**) Immunofluorescence (IF) staining of the phosphorylated NCC (Thr53) in each group. The value of the WT-saline group was set to one and expressed as a fold change. All values are presented as the mean ± SD. Statistical significance was measured using Mann–Whitney test. * *p* < 0.05, compared with the saline-infused Prdx5 WT. ** *p* < 0.05, compared with Ang-II-infused Prdx5 WT.

In this study, Prdx5 KO mice were used to elucidate the role of Prdx5 in Ang-II-induced hypertension. However, there is no report on the pathological condition with complete Prdx5 deficiency. Since the condition with reduced Prdx5 is clinically more reliable, we re-evaluated the role of Prdx5 in Ang-II-induced hypertension using Prdx5 hetero mice. Under the same condition as Prdx5 KO, the same dose of Ang II (1000 ng/kg/day) was infused into Prdx5 hetero mice for 4 weeks. Blood pressure was observed to be increased in Ang-II-infused Prdx5 hetero mice compared to Ang-II-infused WT mice (Figure 8A). However, unlike the results in Prdx5 KO mice, the ratio of kidney weight to body weight (KW/BW), sCr, and BUN did not show significant differences in Ang-II-induced Prdx5 hetero mice compared to Ang-II-induced Prdx5 WT (Figure 8B–D). Additionally, FENa (%), which represents the ratio of Na filtered through the glomerulus and excreted in the urine without being reabsorbed, was more reduced in Ang-II-infused Prdx5 hetero than in Ang-II-infused Prdx5 WT. This explained the phenomenon of greater sodium retention in Ang-II-infused Prdx5 hetero mice (Figure 8E). Expression of renal fibrotic markers (α-SMA, vimentin and fibronectin) also increased in the kidneys of Ang-II-infused Prdx5 hetero mice with reduced Prdx5 (Figure 8F). The expression of the activated form of the WNK4-SPAK/OSR1-NCC axis was also increased in Ang-II-infused Prdx5 hetero, similar to the results in Prdx5 KO mice (Figure 8G,H). These results suggest that the reduction of Prdx5 is a major pathogenic factor in the elevation of blood pressure by Ang II, and the mechanism is due to the increased activation of NCC.

## 4. Discussion

**Figure 9:**
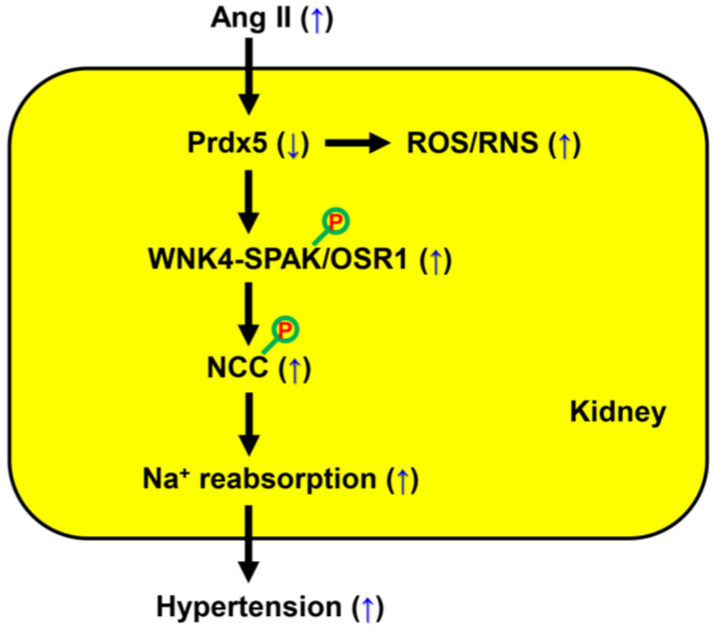
A schematic diagram. In summary, Ang II acts as a key mediator of hypertension. The decrease in Prdx5 by Ang II in the kidney is one of the causes of hypertension, and its mechanism of action was related to the regulation of the activation of the WNK4-SPAK/OSR1-NCC axis. In Prdx5 KO mice lacking Prdx5, the activation of the WNK4-SPAK/OSR1-NCC axis, which plays an important role in sodium reabsorption in the kidney, was increased by Ang II infusion. The loss of Prdx5 resulted in increased production of ROS/RNS such as superoxide and peroxinitrite, and the production of ROS/RNS is regulated depending on the expression level of Prdx5. In conclusion, Prdx5 has a role in negatively regulating the activity of the WNK4-SPAK/OSR1-NCC axis in the kidney. This result suggests that it may be a target for future therapeutics as an inhibitor of NCC. Activation by phosphorylation is denoted with a red ‘P’.

The link between hypertension and CKD is complex and multifactorial, involving different mechanisms. These pathogenic mechanisms include sodium dysregulation, increased sympathetic nervous system activity, and alterations in the renin–angiotensin–aldosterone system. Medical treatment of CKD-related hypertension is complex, and the development of therapeutic agents is challenging. In this study, we aimed to elucidate whether Prdx5 plays a role in the pathogenesis of CKD-related hypertension and the molecular regulatory mechanisms by Prdx5 in the kidney. Under Ang II infusion conditions, Prdx5 KO mice were not only vulnerable to renal damage, including increased pathological characteristics of renal fibrosis, but also showed a worsening of Ang-II-induced blood pressure elevation than Ang-II-infused WT mice (Figure 1C and Figure 4). However, in the absence of Ang II infusion, the blood pressure of Prdx5 KO mice was not significantly different from that of WT mice (Figure 1C). In addition, we confirmed that Prdx5 KO mice showed a marked increase in the expression of Prdx4 among Prdx isotypes in the kidney (Figure 1D). Recently, Prdx4 has been reported to regulate oxidative stress and inflammation through interaction with the dopamine D5 receptor, which is related to blood pressure regulation [27]. In the kidneys of Prdx5 KO mice, the expression of renal fibrosis markers, such as SMA, vimentin, FN, TGF-β, and CTGF, was already slightly increased compared to Prdx5 WT (Figure 4). Taken together, Prdx5 deficiency itself is not a sufficient prerequisite for hypertension, but it is a sufficient condition for the progression of renal fibrosis. Furthermore, when stimulated with Ang II, Prdx5 deficiency is sensitive to the induction of hypertension. In particular, in the kidney, the production of free radicals such as superoxide anion and peroxynitrite by Ang II was increased (Figure 3). Although it has not been clearly identified whether the activity of the WNK4-SPAK/OSR1-NCC axis is directly regulated by ROS/RNS, Prdx5 played a role as an antioxidant protein that fine tunes ROS/RNS generated by Ang II. The activity of NCC, which exists in the distal convoluted tubule and is involved in sodium retention, was subsequently increased by the activity of WNK4-SPAK/OSR1. Finally, it is suggested that the decrease in Prdx5 by Ang II is one of the leading causes of hypertension (Figure 9).

In both Prdx5 KO and Prdx5 hetero mice, the increase in blood pressure induced by Ang II was worse than in Prdx5 WT. Furthermore, the activation of NCC occurred as the causal mechanism of the increase in blood pressure in both mice. In renal pathology, a situation like Prdx5 KO mouse, where Prdx5 is completely absent, has not been reported yet. Rather, there is a report that the decrease in Prdx5 expression is associated with renal disease. For example, protein levels of Prdx5 were decreased in autosomal dominant polycystic kidney disease (ADPKD) [28], I/R-induced acute kidney injury [21], UUO-induced renal fibrosis [22,23], etc. When ectopic overexpression of Prdx5 was performed, this pathophysiological phenomenon was alleviated [21,22,23,28]. The results obtained using Prdx5 hetero mice can be considered to better represent the pathological situation that can occur clinically. Although the mechanism by which Prdx5 is reduced by Ang II has not yet been elucidated, it can be known that Prdx5 physiologically plays a role in protecting the kidney from disease. Ang II induces hypertension primarily through its response to AT1 receptors in the kidney [29]. AT1 receptors are widely distributed throughout nephron segments, including the proximal tubule, thick ascending limb of the loop of Henle, macula densa, distal tubule, and collecting duct (CD) [30]. In this study, Ang-II-induced Prdx5 KO or Prdx5 hetero activated NCC regulation in the distal tubule. Future studies would be needed to generate distal-tubule-specific conditional KO mice [31] and investigate how Prdx5 regulates the WNK4-SPAK/OSR1-NCC axis.

Activation of the WNK4-SPAK/OSR1-NCC signaling axis is one of the master signal pathways responsible for hypertension. The previously reported results suggested seven therapeutic strategies to control the activity of this axis [19], which include (1) direct inhibition of NCCs by thiazide diuretics [32,33], (2) allosteric or orthosteric inhibition of WNK kinases [34,35], (3) inhibition of SPAK/OSR1 [36,37], (4) inhibition of mouse protein-25 (MO25) [38], (5) inhibition of the WNK-SPAK/OSR1 interaction or the SPAK/OSR1-NCC interaction [39,40], (6) stabilization of the CUL3/KLHL3 interaction [14,41,42], and (7) inhibition of the ubiquitination and glycosylation of NCC [43,44]. In this study, in Prdx5 downregulated 209/mDCT cells, phosphorylation of SPAK/OSR1 and phosphorylation of NCC were increased (Figure 7); however, when Prdx5 was overexpressed, the phosphorylation of SPAK/OSR1 and phosphorylation of NCC were decreased (Figure 6). We observed that Prdx5 effectively regulated the activation of the WNK4-SPAK/OSR1-NCC signaling axis. However, the mechanism by which Prdx5 regulates the activity of the WNK4-SPAK/OSR1-NCC signaling axis remains unknown. In future studies, it will be necessary to study whether the function of Prdx5 is related to the regulatory mechanism at the seven therapeutic strategy sites or whether it is through a mechanism that has not yet been reported. Therefore, effective modulation in WNK-SPAK/OSR1-NCC axis of Prdx5 might provide an important therapeutic strategy for anti-hypertensive agents.

## 5. Conclusions

In conclusion, chronic stimulation of Ang II induced a decrease in renal Prdx5, resulting in ROS/RNS production, and accelerated the progression of renal fibrosis, a hallmark of CKD. In particular, the decrease in Prdx5 by Ang II in DCT increased the activity of the WNK4-SPAK/OSR1-NCC axis associated with the development of hypertension. This response is correlated with the ROS/RNS level according to the expression level of Prdx5. A lack of Prdx5 is responsible for the pathogenesis of CKD, hypertension. Therefore, we suggest that Prdx5 could be one of the prime targets for treating CKD and NCC-associated hypertension.

## Data Availability

Data are contained within the manuscript and Supplementary Materials.

## Supplementary Materials

The following supporting information can be downloaded at: https://www.mdpi.com/article/10.3390/antiox14010100/s1; Figure S1: Expression level of Prdx5 in various tissues of Prdx5 KO mice; Table S1: Mouse qPCR primer sequences.

## Abbreviations

Ang II	Angiotensin II
BUN	Blood urea nitrogen
CKD	Chronic kidney disease
CPH	1-Hydroxy-3-methoxycarbonyl-2,2,5,5-tetramethylpyrrolidine. HCl
DCT	Distal convoluted tubule
DHE	Dihydroethidium
DMPO	5,5-Dimethyl-1-pyrroline-N-oxide
ECF	Extracellular fluid
EPR	Electron paramagnetic resonance
FENa	Fractional excretion of sodium
HHE	4-hydroxy-2 hexenal
I/R	Ischemia/reperfusion
KO	Knock-out
NCC	Sodium-chloride cotransporter
OSR1	Oxidative-stress-responsive kinase 1
PAS	Periodic acid–Schiff
Prdx5	Peroxiredoxin 5
RAS	Renin–angiotensin system
RNS	Reactive nitrogen species
ROS	Reactive oxygen species
sCr	Serum creatinine
sNGAL	Serum neutrophil gelatinase-associated lipocalin
SNS	Sympathetic nervous system
SPAK	STE20/SPS1-related proline/alanine-rich kinase
TGF-β	Transforming growth factor-β
WNK4	With No lysine (K) protein kinase 4
WT	Wild-type
α-SMA	alpha-smooth muscle actin
