# Supplementary material for: Peroxiredoxin 5 Acts as a Negative Regulator of the Sodium-Chloride Cotransporter Involved in Alleviating Angiotensin II-Induced Hypertension
Source: Antioxidants (Basel). 2025 Jan 16;14(1):100. doi: 10.3390/antiox14010100 (PMC11761572; doi:10.3390/antiox14010100)
Supplement: Supplementary file 1 [file antioxidants-14-00100-s001.zip › antioxidants-3415836-supplementary.pdf]

Supplementary Materials

Peroxiredoxin 5 acts as a negative regulator of the sodium chloride cotransporter involved in alleviating angiotensin II-induced hypertension

Hoon-In Choi[1], In Ae Jung[1], and Soo Wan Kim[1]*

[1] Department of Internal Medicine, Chonnam National University Medical School & Hospital, Gwangju 61469, Korea; skimw@chonnam.ac.kr

*Correspondence: skimw@chonnam.ac.kr; Tel.: +82 62 220 6271

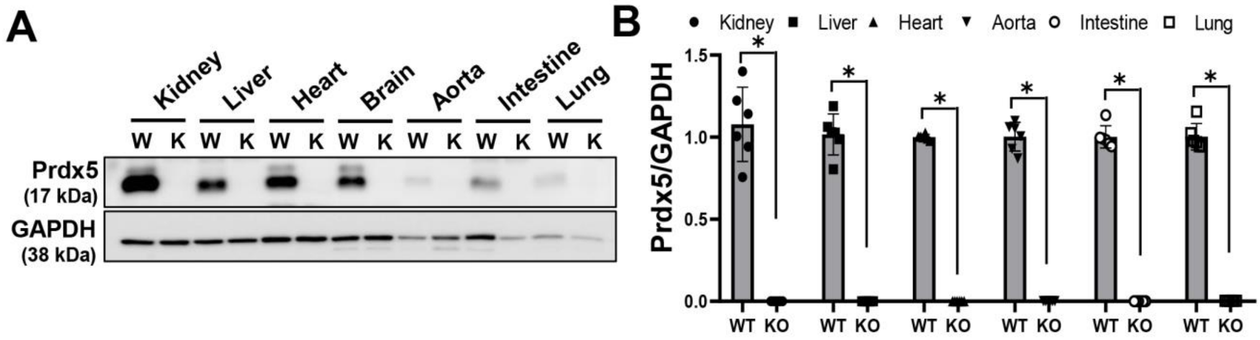

Figure S1. Expression level of Prdx5 in various tissues of Prdx5 KO mice. In WT mice and Prdx5 KO mice, the protein level (A) and mRNA level (B) of Prdx5 was confirmed by western blotting and real-time PCR in the kidney, liver, heart, brain, aorta, intestine, and lung tissues. GAPDH was used as an internal control. For bar grapes, the value of the WT_Saline group was set to one and expressed as a fold change. All values are presented as the mean ± SD. Statistical significance was measured using Mann-Whitney test. *$p < 0.05$, compared with the saline-infused Prdx5 WT. **$p < 0.05$, compared with Ang II-infused Prdx5 WT.

Table S1. Mouse qPCR primer sequences

Gene	RefSeq accession number	Forward primer	Reverse primer
Prdx5	NM_012021	5′-TTATTGGATGATTCTTTGGTC-3′	5′-CCTTCACTATGCCGTTGTCTATCAC-3′
α-SMA	NM_007392.3	5′-CTGACAGAGGCACCACTGAA-3′	5′-AGAGGCATAGAGGGACAGCA-3′
Vimentin	NM_011701.4	SinoBiological, Cat # MP200442	
Fibronectin	NM_010233	5′-ACAGAGCTCAACCTCCCTGA-3′	5′-TGTGCTCTCCTGGTTCTCCT-3′
CTGF	NM_010217.2	5′-CACAGAGTGGAGCGCCTGTTC-3′	5′-GATGCACTTTTTGCCCTTCTTAATG-3′
TGF-β	NM_011577.2	5′-CAGGAGCGCACAATCATGTT-3′	5′-CTTTAGGAAGGACCTGGGTT-3′
GAPDH	NM_001289726	5′-TGTGTCCTCGTGGATCTGA-3′	5′-GATGCCTGCTTCACCACCTT-3′
